# A neonate with late-onset hypocalcemia due to unrecognized maternal hyperparathyroidism and a systematic overview of similar cases

**DOI:** 10.3205/000296

**Published:** 2021-07-28

**Authors:** Georgios Mitsiakos, Georgios N. Katsaras, Ilias Chatziioannidis, Anastasia Gkampeta, Christina Mitsiakou, Nikolaos Nikolaidis

**Affiliations:** 1 2nd Neonatal Department and Neonatal Intensive Care Unit, Aristotle University of Thessaloniki, “Papageorgiou” General Hospital of Thessaloniki, Greece

**Keywords:** neonates, hypocalcemia, hyperparathyroidism, seizures

## Abstract

**Objective:** Neonatal seizures are alarming manifestations of an underlying significant disorder demanding immediate attention and intervention. Hypocalcemia, although rare, must be considered in the differential diagnosis of neonatal seizures.

**Method:** We present an unusual case of a 10-day-old infant with unexplained symptomatic hypocalcemia, experiencing multiple episodes of focal tonic-clonic seizures, born by an entirely asymptomatic mother. Moreover, we conducted a systematic search in PubMed and Scopus databases to present a clinical overview of all similar cases.

**Result:** Maternal laboratory investigation revealed markedly increased calcium levels with concomitant high parathyroid hormone levels due to a parathyroid adenoma, undiagnosed during antenatal checkup.

**Conclusion:** This is one of the few cases in the literature where neonatal symptomatology led to the diagnosis of undiagnosed maternal hyperparathyroidism. Early detection and appropriate management of neonatal hypocalcemia could eliminate serious maternal and fetal morbidity.

## Zusammenfassung

**Zielsetzung:** Krampfanfälle bei Neugeborenen sind alarmierende Manifestationen einer zugrunde liegenden signifikanten Störung, die sofortige Aufmerksamkeit und Intervention erfordert. Obwohl Hypokalzämien selten sind, müssen sie bei der Differentialdiagnose von Krampfanfällen bei Neugeborenen berücksichtigt werden.

**Methode:** Wir präsentieren einen ungewöhnlichen Fall eines 10 Tage alten Kindes mit ungeklärter symptomatischer Hypokalzämie, bei dem mehrere Episoden fokaler tonisch-klonischer Anfälle auftraten. Das Kind wurde von einer völlig asymptomatischen Mutter geboren. Darüber hinaus haben wir eine systematische Suche in den PubMed- und Scopus-Datenbanken durchgeführt, um einen klinischen Überblick über alle ähnlichen Fälle zu erhalten.

**Ergebnis:** Laboruntersuchungen bei der Mutter ergaben einen deutlich erhöhten Kalziumspiegel bei gleichzeitig hohen Nebenschilddrüsenhormonspiegeln aufgrund eines Nebenschilddrüsenadenoms, das während der vorgeburtlichen Untersuchungen nicht diagnostiziert wurde.

**Schlussfolgerung:** Dies ist einer der wenigen Fälle in der Literatur, in denen die Neugeborenen-Symptomatik zur Diagnose eines nicht diagnostizierten mütterlichen Hyperparathyreoidismus führte. Eine frühzeitige Erkennung und angemessene Behandlung der neonatalen Hypokalzämie könnte eine schwerwiegende Morbidität bei Mutter und Kind verhindern.

## Introduction

Neonatal hypocalcemia, although rare, must be considered in the differential diagnosis of neonatal seizures which are alarming manifestations of an underlying disorder demanding immediate evaluation and intervention [1]. Neonatal hypocalcemia occurring in the first three days of life is defined as early-onset, whereas occurring after the first three days of life it is defined as late-onset hypocalcemia [2].

Primary hyperparathyroidism (PHP) during pregnancy is a very rare condition increasing both maternal and perinatal morbidity and mortality. Thus, early detection and intervention is mandatory, the optimal time being during the second trimester [3], [4], [5], [6]. Maternal hyperparathyroidism can lead to profound neonatal hypocalcemia and hypocalcemic tetany, occurring approximately in 50% of infants born from mothers with untreated disease. Neonatal hypocalcemia is attributed to prolonged parathyroid suppression from the chronic hypercalcemic maternal state and the abrupt halt of maternal calcium following delivery [7].

The investigation of a newborn’s hypocalcemia should not focus on the patient alone. Detailed examination and laboratory investigation of an apparently healthy mother could be beneficial for both neonate and mother.

In this article, we present a case of neonatal seizures due to undiagnosed maternal hyperparathyroidism. What is more, we conducted a systematic search in PubMed and Scopus databases using the terms “maternal hyperparathyroidism”, “hyp?parathyroidism”, “neonat*” and “newborn” in order to meta-analyze the published case reports of neonatal events that led to the diagnosis of unrecognized maternal hyperparathyroidism.

## Case description

A 10-day-old male neonate was admitted to our Neonatal Intensive Care Unit (NICU) due to multiple episodes of focal tonic-clonic seizures. He was born at 40 weeks gestational age and 3060 g birth weight after an uneventful pregnancy and an uncomplicated vaginal delivery. The last 24 hours, he started having several episodes of left upper extremity’s jerking combined with rapid eye blinking on the same side. There was no history of trauma or clinical signs of infection.

On admission, a detailed clinical examination and a full sepsis screen, including lumbar puncture, revealed no signs of infection. Detailed laboratory investigation exhibited hypocalcemia with hyperphosphatemia [Ca=5.4mg/dl (normal reference range: 9.0–10.9 mg/dl), P=11.8mg/dl (normal reference range: 4.8–8.2 mg/dl)]. Immediately, Ca gluconate 10% (8 ml/H) was administered to the infant, but seizures did not resolve, hypocalcemia persisted while the neonate experienced an episode of bradycardia.

A subsequent brain ultrasound and cerebral function monitoring (CFM) were negative for cerebral pathology. Serum electrolytes, plasma glucose and arterial gases were all within normal ranges. However, total serum and ionized calcium levels were low [7.4 mg/dl and 3.8 mg/dl (normal reference range: 4.52–5.2 mg/dl), respectively), with normal magnesium and high phosphate levels (1.6 mg/dl and 10.2 mg/dl, respectively). Parathyroid hormone (PTH) levels were low (14.5 pg/ml, normal reference range: 10–65 pg/ml).

The neonate started calcium gluconate 10% (8ml/H) as well as vitamin D (1a-OH D3, 0.25 µg/day), and monitoring of PTH and serum electrolytes was established. The seizures resolved after 24 hours and the neonate was discharged 20 days later with normal electrolyte levels. Vitamin D and calcium supplement were stopped after 3 months. During follow-up, the patient remained seizure-free, with normal serum calcium and PTH levels (9.66 mg/dl and 22.3 pg/ml).

Because of the unexplained neonatal hypocalcemia secondary to hypoparathyroidism, a maternal blood sample was taken for further evaluation. The mother was clinically asymptomatic. She had a positive history of nephrolithiasis from a few years ago. The mother’s evaluation revealed high total calcium and ionized calcium levels (12.48 mg/dl and 7 mg/dl, respectively) with concomitant high PTH (201 pg/ml) levels. Based on these findings, maternal hyperparathyroidism was suspected. Further investigation with ultrasonography revealed a hypoechogenic nodule in the thyroid gland, and Tc-99m sestamibi scintigraphy demonstrated focally increased uptake in the corresponding site, suggestive of parathyroid adenoma. After 6 months, a macroscopically nodule of 0.5 g weight and 2 cm maximum diameter was removed surgically, while the histopathologic examination confirmed the diagnosis of parathyroid adenoma.

## Discussion

We describe a rare case of neonatal symptomatology leading to the diagnosis of maternal hyperparathyroidism. The case underlines the necessity of maternal investigation whenever neonatal hypocalcemia is detected.

Maternal hypercalcemic hyperparathyroidism causes suppression of fetal parathyroid glands secondary to an increased calcium flux across the placenta to the fetus. This leads to neonatal hypoparathyroidism and subsequent hypocalcemia. This could be attributed to the unknown interaction of parathyroid hormone-related peptide (PTHrP) that could activate the PTH receptor resulting in hypercalcemic and hyperphosphaturic PTH-like activity during the early neonatal period. Although its detailed physiological function remains unknown, PTHrP could play a role during pregnancy and the perinatal period as it is produced by the placenta and fetal parathyroid glands and is found in the mother’s milk as well [7], [8].

Primary maternal hyperparathyroidism is a rare but severe complication of pregnancy. The early diagnosis during pregnancy remains difficult, as the symptoms are usually subtle and are easily confused with other minor complications of pregnancy. Maternal symptomatology may be nonspecific with fatigue, subtle mental changes, weakness, and hyperemesis; or more specific with nephrolithiasis, pancreatitis, gastrointestinal ulcers, and life-threatening hypercalcemic crisis. Rarely, signs of complicated gestation are present (intrauterine growth retardation, spontaneous abortion, preterm delivery, low birth weight) [8], [9], [10], [11], [12], [13], [14], [15]. In our case, the mother had a renal colic due to nephrolithiasis 2 years ago, treated with medications without recurrences.

**Figure 1:**
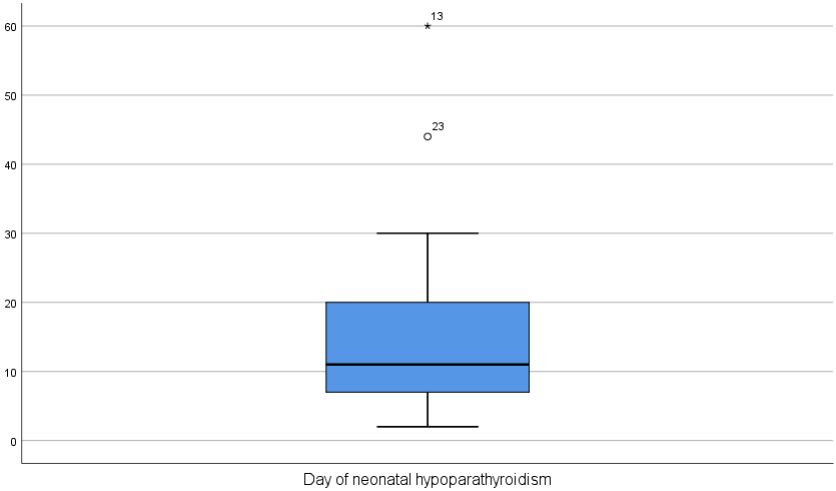
Box-Plot: Day of neonatal clinical findings of hypoparathyroidism

Worldwide, few cases of neonatal hypoparathyroidism due to maternal unrecognized hyperparathyroidism have been described (Table 1 (Tab. 1)). Since 1938, 41 cases have been described [16], [17], [18], [19], [20], [21], [22], [23], [24], [25], [26], [27], [28], [29], [30], [31], [32], [33], [34], [35], [36], [37], [38], [39], [40], [41], [42], [43], [44], [45], [46], [47]. A qualitative analysis of these cases showed that the median day of neonatal hypoparathyroidism is the 11^th^ postnatal day with IQR: 7^th^–21^st^ postnatal days (Figure 1 (Fig. 1)). The majority of cases presented with seizures (63.4%), followed by tetanus (19.5%). Other symptoms, such us twitching, choking, pitch crying, irritability, dyspnea, severe metabolic disease and circulatory collapse were rarely reported (2.4% each of them).

## Conclusions

Our case enhances the findings of the current literature. As neonatal hypocalcemia usually occurs soon after birth in high-risk infants, any unexplained late-onset hypocalcemia, especially if neonatal seizures are present, warrants further investigation including maternal calcium and phosphate levels. High obstetricians’ and pediatricians’ alertness are required for early detection of maternal hyperparathyroidism and its underlying cause.

## Notes

### Informed consent

Informed consent has been obtained from the patient’s mother for the publication of this case report.

### Authors’ contributions

Georgios Mitsiakos participated in supervision, conceptualization, investigation, methodology, writing, original drafting of the manuscript and validating the final form.

Georgios N. Katsaras, Anastasia Gkampeta and Christina Mitsiakou participated in conceptualization, investigation, methodology, writing and original drafting the manuscript. Ilias Chatziioannidis, Paraskevi Karagianni and Nikolaos Nikolaidis were responsible for investigation, methodology, validation, editing and reviewing the paper.

### Competing interests

The authors declare that they have no competing interests.

## Figures and Tables

**Table 1:**
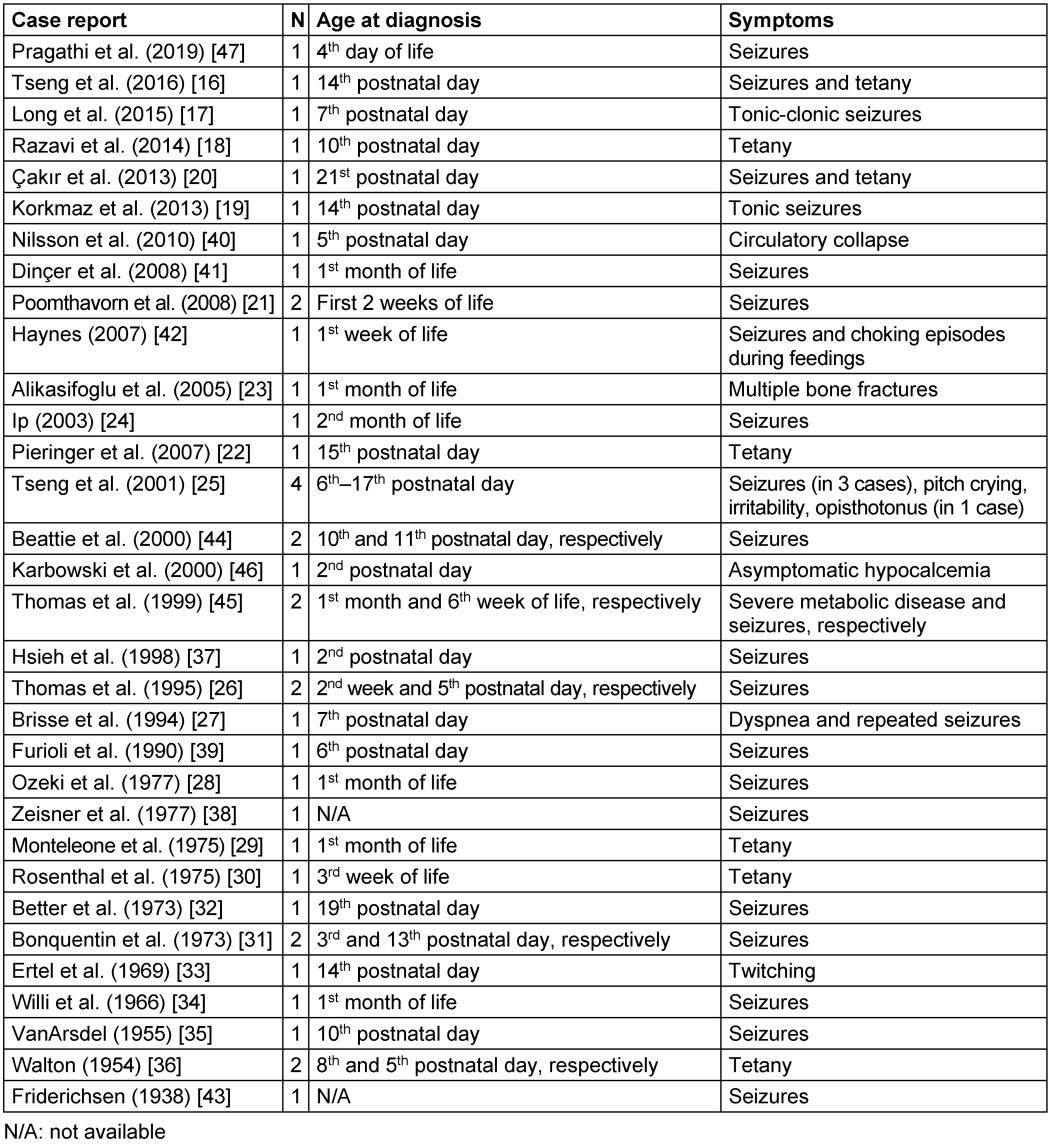
Published cases of neonatal hypoparathyroidism due to maternal unrecognized hyperparathyroidism from 1938 until today
